# Influence of proportion of Brahman genetics on productivity of Brahman–Angus cows at weaning

**DOI:** 10.1093/tas/txae093

**Published:** 2024-06-06

**Authors:** Thiago Martins, Cecília Constantino Rocha, Joseph Danny Driver, Owen Rae, Mauricio Aguirre Elzo, Raluca G Mateescu, José Eduardo Portela Santos, Mario Binelli

**Affiliations:** Division of Animal Sciences, University of Missouri, Columbia, MA, USA; Department of Animal Sciences and D.H. Barron Reproductive and Perinatal Biology Research Program, University of Florida, Gainesville, FL, USA; Department of Animal Sciences and D.H. Barron Reproductive and Perinatal Biology Research Program, University of Florida, Gainesville, FL, USA; Department of Large Animal Clinical Sciences, University of Florida, FL, USA; Department of Animal Sciences and D.H. Barron Reproductive and Perinatal Biology Research Program, University of Florida, Gainesville, FL, USA; Professor Emeritus, Department of Animal Sciences, University of Florida, FL, USA; Department of Animal Sciences and D.H. Barron Reproductive and Perinatal Biology Research Program, University of Florida, Gainesville, FL, USA; Department of Animal Sciences and D.H. Barron Reproductive and Perinatal Biology Research Program, University of Florida, Gainesville, FL, USA; Department of Animal Sciences and D.H. Barron Reproductive and Perinatal Biology Research Program, University of Florida, Gainesville, FL, USA

**Keywords:** Beef cattle, *Bos indicus*, calf performance, weaning, tropics

## Abstract

This study evaluated the association between the proportion of Brahman genetics and productivity of Brahman–Angus cows at weaning using a 31-yr dataset containing 6,312 cows and 5,405 pregnancies. Cows were contemporaneously reared and enrolled in yearly breeding seasons under subtropical conditions of North-Central Florida. They were evenly distributed in six-breed groups (G) according to the proportion of Brahman genetics: G0% to 19%, G21% to 34%, G38% (Brangus), G41% to 59%, G63% to 78%, and G81% to 100%. The proportion of cows calving (84.9%) did not differ across the six-breed groups. However, cows in the G81% to 100% weaned fewer calves (90.8%) than cows in the G0% to 19% and G21% to 34% (95.7%, each). The weaning rate of cows in the G38% (94.3%), G41% to 59% (94.2%), and G63% to 78% (93.0%) was intermediate between these three breed groups. The preweaning calf mortality was greater for cows in the G81% to 100% (9.2%) than cows in the G0% to 19% and G21% to 34% (4.3%, each), but intermediate for cows in the G38% (5.7%), G41% to 59% (5.8%), and G63% to 78% (7.0%). Cows in the G81% to 100% also weaned lighter calves (220.6 kg) than cows in the G0% to 19% (245.2 kg), G21% to 34% (250.2 kg), G38% (247.9 kg), G41% to 59% (252.5 kg), and G63% to 78% (245.2 kg). Cows in the G0% to 19% weaned lighter calves than cows with 21% to 78% of Brahman genetics. The 205-d adjusted weaning weight evidenced the less productive results of cows in G0% to 19% and G81% to 100% compared with other genetic groups, as they calved at the fastest and slowest rate, respectively. Thus, the 205-d adjusted weaning weight eliminated this bias. Additionally, younger cows weaned lighter calves; and male calves were heavier at weaning than female calves. Both parity order of cow and calf sex altered the magnitude of the described association between breed group of cows and calf weaning weights. Overall, after adjusting for weaning rate and age of calves at weaning, the number of kilograms produced per cow submitted to reproduction was less for cows in the G0% to 19% (191.1 kg) and G81% to 100 (181.8 kg) compared with cows in the G21% to 34 (197.0 kg), G38 (195.9 kg), G41% to 59 (199.7), and G63% to 78 (196.2). Cows in the G81% to 100% were the least productive. Thus, a proportion of Brahman genetics between 21% and 78% ensured superior productivity of Brahman–Angus cows subjected to subtropical conditions.

## Teaser

Crossbreeding between *Bos taurus* and *Bos indicus* breeds is a common strategy used in tropical and subtropical regions to enhance beef production. However, the extent of the *B. indicus* influence on kilograms of calf produced per cow submitted to reproduction remains uncharacterized. In this study, we analyzed the productivity of Brahman–Angus cows bearing 0% to 100% of Brahman genetics reared contemporaneously. We verified that Brahman–Angus cows with proportion of Brahman genetics ranging from 21% to 78% were among the most productive.

## Introduction

*Bos indicus* and crosses between *B. indicus* and *Bos taurus* breeds are predominant in subtropical and tropical regions. Tropical regions, which are characterized by greater temperature, humidity, incidence of parasitic and parasite-transmitted disease, and forages that often have less nutritive value compared to temperate regions. These harsh conditions usually result in reduced growth rate and reproductive performance of *B. taurus* breeds (Burrow, 2015). Although *B. indicus* breeds are more resilient to these stressors than the *B. taurus* breeds (Rechav, 1987; Hansen, 2004), they display inferior productive traits such as reduced feed efficiency, late reproductive maturity, and leaner beef (Hruska et al., 1993; Casas et al., 2011; Cooke et al., 2020). Crossbreeding *B. indicus* and *B. taurus* cattle is a common strategy to overcome undesirable traits and enhance beef production in tropical and subtropical areas (Lamy et al., 2012). The superiority of crossbred animals is attributed to the combination of traits from complementary breeds through gene additive effect and heterosis (Elzo and Wakeman, 1998; Leal et al., 2018). Indeed, the heterosis effect results in gains of productivity that include increases in weaning weight and milk yield (Syrstad, 1985; Casas et al., 2011). However, there are limited studies in the literature that evaluated the association between the proportion of *B. indicus* genetics of crossbred animals and productivity during the cow–calf phase of the beef production cycle. The previous and complementary article to the present study evaluated this relationship from the reproductive point of view using a 31-yr accumulated multibreed herd population (Brahman × Angus) dataset from the University of Florida (Martins et al., 2022). Herein, the productivity of these same Brahman–Angus cows was followed up to weaning. Productivity was defined as kilograms of calf weaned divided by the number of cows submitted to reproduction. The overarching objective was to determine the relationship between the proportion of Brahman genetics of Brahman–Angus cows and their productivity at weaning. To assess productivity, analyses included the estimation of calving rates, risk of stillbirth, risk of calf mortality, weaning rate, and weaning weight of calves according to the proportion of Brahman genetics of these cows. The hypothesis tested was that a moderate proportion of Brahman genetics ensures the greatest productivity of Brahman–Angus cows managed under subtropical conditions.

## Material and Methods

### Animals and calving season

Productivity of Brahman–Angus cows submitted to reproduction was assessed at weaning according to the proportion of Brahman genetics of cows. The reproductive performance of these cows was presented in Martins et al. (2022).

Productivity was defined as kilograms of calf weaned divided by the number of cows submitted to reproduction. Thus, productivity was a compound result from losses due to failure to become pregnant, failure to calve, stillbirth, deaths of cows or calves, and the weaning weight of calves. Brahman–Angus cows, from the University of Florida multibreed herd (Elzo and Wakeman, 1998), were identified to each 1/32nd fraction (approximately 3-percentage points of Brahman genetics) and analyzed in six-breed groups according to the proportions of Brahman: G0% to 19%, G21% to 34%, G38% (Brangus), G41% to 59%, G63% to 78%, and G81% to 100%. This multibreed herd population was at research stations located in North-Central Florida in the city of Gainesville (latitude 29°39’55” N; longitude 82°20’10” W). Thus, animals were under a humid subtropical climate. Because data were obtained from a preexisting database, there was no need for Institutional Animal Care and Use Committee (IACUC) approval.

The experimental data was collected from 1989 to 2020 for a total of 31 breeding and calving seasons. Response variables retrieved included reproductive, calving, and weaning measurements. Details about feeding and reproductive management of this herd were described in Martins et al. (2022). Briefly, the breed groups were managed contemporaneously, in allotments, under grazing conditions (*Paspalum notatum*, *Cynodon dactylon*, or *Lolium*) with ad libitum access to water and minerals to meet or exceed maintenance requirements. During late fall and early winter, cows were fed hay or haylage and supplements that included mixtures of soybean hulls, whole cottonseed, corn gluten feed, and citrus pulp, according to the availability, cost, and nutritional needs of the cows. In the middle of winter, cows grazed winter cultivated rye cereal (*Secale cereale*) or ryegrass (*Lolium*) pastures. Breeding events occurred between late winter and summer. Throughout the years, cows were submitted to different estrous synchronization programs for artificial insemination before being serviced by clean-up bulls. Nulliparous cows were submitted to reproduction 15 to 21 d earlier than every other parity category order.

The balance on the proportion of Brahman and Angus influence into the herd was maintained by the diallel crossbreeding scheme, in which the six sire groups were reciprocally mated with the six cow groups (Komender, 1988; Elzo and Wakeman, 1998). In the case of artificial insemination, semen from non-commercialized sires was collected and frozen by third-party reproductive companies and evenly assigned across the six-breed group of cows during artificial insemination. These non-commercialized semen straws were stored and handled exactly as commercial semen straws. The calf crop was a representative sample of the six genetic groups. Most calves were born between November and March (*n* = 5,295), and only 69 calves were born between April and May. The weaning occurred in 2 d during the months of August, September, or early October. At weaning, the mean (±SD) age of calves was 231.5 ± 28.7 d, ranging from 120 to 309 d.

### Data and records

Individual data from each cow–calf pair were retrieved from historical records in Excel spreadsheets and compiled for analyses. In Martins et al. (2022), we analyzed the reproductive performance of cows throughout the years of 1989 to 2020, except for the year 2001 because of the incompleteness of the data; totalizing 31 breeding seasons analyzed. Here, the dataset used in Martins et al. (2022), containing the information of pregnancies generated during the breeding season, was complemented with the dataset generated during the corresponding subsequent calving season. Traceability was possible because calves were tagged, and the cow–calf pair was identified at birth. Each cow and calf had a unique identification number. The working spreadsheet was complemented with the following information about the calves: birth date, birth weight, calf sex, disposal comments (such as sale, euthanasia, stillbirth, and deaths), weaning date, and weaning weight. Information on twinning and freemartin incidence were nevertheless missing and assumed to be equally distributed across the six-breed groups and parity orders. In addition, the unidentified twinning or freemartin events should not have had an impact on the results due to the large dataset. In Martins et al. (2022), 6,504 cows generated 5,597 pregnancies (86.1%) after an approximately 90-d breeding season, regardless of the breed group. In the present study, data from 192 pregnancies (G0% to 19%: *n* = 36, G21% to 34%: *n* = 46, G38%: *n* = 24, G41% to 59%: *n* = 40, G63% to 78%: *n* = 20, and G81% to 100%: *n* = 26) were disregarded because cows were sold before calving. Thus, a total of 6,312 cows and 5,405 pregnancies were considered for analyses. The individual sire identification name was not always available or neatly recorded in older records, despite the breed group of sires being properly recorded for determination of breed composition of calves. Cows were classified according to four parity orders at the breeding season: nulliparous (2.1 ± 0.08 yr of age, mean ± SD; range from 1.8 to 2.3), primiparous (3.2 ± 0.09; 2.8 to 3.5), secundiparous (4.2 ± 0.09; 3.9 to 4.5), and pluriparous cows (7.2 ± 2.2; 4.0 to 16.5). Across the six-breed groups, the average year of age was G0% to 19%: 5.4 ± 2.9 (1.9 to 16.2), G21% to 34%: 4.7 ± 2.5 (1.9 to 14.2), G38%: 4.7 ± 2.6 (1.9 to 15.1), G41% to 59%: 4.8 ± 2.6 (1.8 to 15.2), G63% to 78%: 4.3 ± 2.2 (1.8 to 14.2), and G81% to 100%: 4.4 ± 2.3 (1.9 to 15.3).

Cow was the experimental unit of analysis for the records retrieved. Each breeding season contributed a mean of 210 records (ranged from 103 to 345 records). Two separate datasets were generated. The first dataset was used to calculate the calf losses between the end of the breeding season and weaning: 1) pregnancy loss = proportion of cows that never calved in a given calving season out of the pregnant cows expected to calve, 2) calving = proportion of calves born out of the total cows exposed to reproduction, 3) stillbirth = proportion of calves born dead out the total calves born, 4) calf mortality = proportion of calves that died between calving and weaning out of the total calves born, and 5) weaning = proportion of calves weaned out of the total cows exposed to breeding. The second dataset was used to evaluate the number of kilograms of calf produced per cow according to the proportion of *B. indicus* genetics of cows considering the non-adjusted weaning weights. Weaning weight of calves was adjusted to 205-d by multiplying the average daily gain from birth to weaning by 205-d and adding the birth weight, as described previously (Elzo and Wakeman, 1998). The calving dates were ordered in reference to the first calving date of each year, which was considered the day 0 (D0) of each calving season. Next, the first calving date was subtracted from each subsequent calving date, resulting in calving periods denoted as D0 to D190. The productivity at weaning was determined by entering the number kilograms of calf weaned, or zero kilogram when a cow did not wean a calf. Then, we calculated the average kilograms of calf produced according to breed group and parity order of cows within each one of the 31 breeding seasons considered, hence totalizing 638 calculated averages (observations) that were used in the statistical analyses for productivity. The number of missing observations (*n* = 106 [744 − 638]) was due to the occasional lack of adequate recording of parity order and/or breed group. Moreover, for each one of the 638 generated observations, we calculated the average age of calf at weaning (120 to 286 d) and weaning rate (0% to 100%) that were included as covariate in one of the statistical models for analysis of productivity.

### Statistical analysis

The statistical analyses were conducted using SAS (version 9.4, SAS Institute Inc., Cary, NC, USA) considering each animal as the experimental unit unless otherwise indicated. The binary-dependent variables (proportion of stillbirth, calf mortality, and calf weaning) were analyzed by logistic regression using generalized linear mixed-effects models with the GLIMMIX procedure of SAS. The continuous-dependent variables (birth weight, non-adjusted weaning weight, 205-d adjusted weaning weight, and productivity) were analyzed by linear mixed-effects models using the MIXED procedure of SAS. The residuals and influencing diagnostics outputs from the MIXED procedure were checked for the assumption of normality of the residuals and homogeneity of variance. In all models, the Kenward–Roger option was used to adjust denominator degrees of freedom to calculate the *F-*values, and only fixed effects that resulted in *P <* 0.20 remained in the final models. In none of the models, the estimation of variance of the effect of cows was possible because the statistical models did not converge when including the random effect of cow because 35.7% of cows were represented only a single year. The sire effect was not considered in the statistical models because not all sires were identified as described previously.

Models for analyses of binary- and continuous-dependent variables included the fixed effects of breed group (G0% to 19%, G21% to 34%, G38%, G41% to 59%, G63% to 78%, or G81% to 100%), parity order (nulliparous, primiparous, secundiparous, or pluriparous), calf sex, and the interactions between breed group and parity order, and breed group and calf sex, and breeding season (1 to 31) as a random effect. For the continuous-dependent variable productivity, the experimental unit was the mean kilograms of calf produced within the 31 breeding seasons (*n* = 638 observations). Productivity was also estimated by adding into the model the variables age of calf at weaning and weaning rate as a covariate for adjustments.

Additional statistical analyses were conducted to determine the associations between days in the calving season when cows calved and weight of calves at birth and weaning. For those, the statistical models included the fixed effects of breed group, calving day (D0 to D190, as a continuous covariate), and the interactions between breed group and calving day, and the random effect of breeding season. Predicted values from mixed-effects models were used to generate the regression curves using Prism GraphPad 9.2.

The interval from the beginning of the calving season and calving was analyzed with the Cox’s proportional hazard regression model using the PHREG procedure of SAS. The variable “time” was the interval in days from the first day of the calving season to the day of calving for each cow. The statistical model included the fixed effects of breed group, parity order, the interaction between breed group and parity order, and breeding season. Cows that did not calve in the calving season were censored on the last day of the respective season or if sold (*n* = 16) or died (*n* = 13) during the calving season, whichever happened first. The adjusted hazard ratios (AHR) were generated using pluriparous and G81% to 100% as reference for parity group and for breed group, respectively. The LIFETEST procedure of SAS was used to calculate the median and mean interval to calving and to generate the survival plots.

Significance was set at *P *≤ 0.05, and tendencies at 0.05 < *P *≤ 0.10. Results are reported as LSMEANS ± SEM for binary- and continuous-dependent variables. Pairwise multiple comparisons were performed with the adjustment by the method of Tukey to determine the difference among LSM.

## Results

### Association between the proportion of Brahman genetics of Brahman–Angus cows and calving distribution

**Fig. 1.**
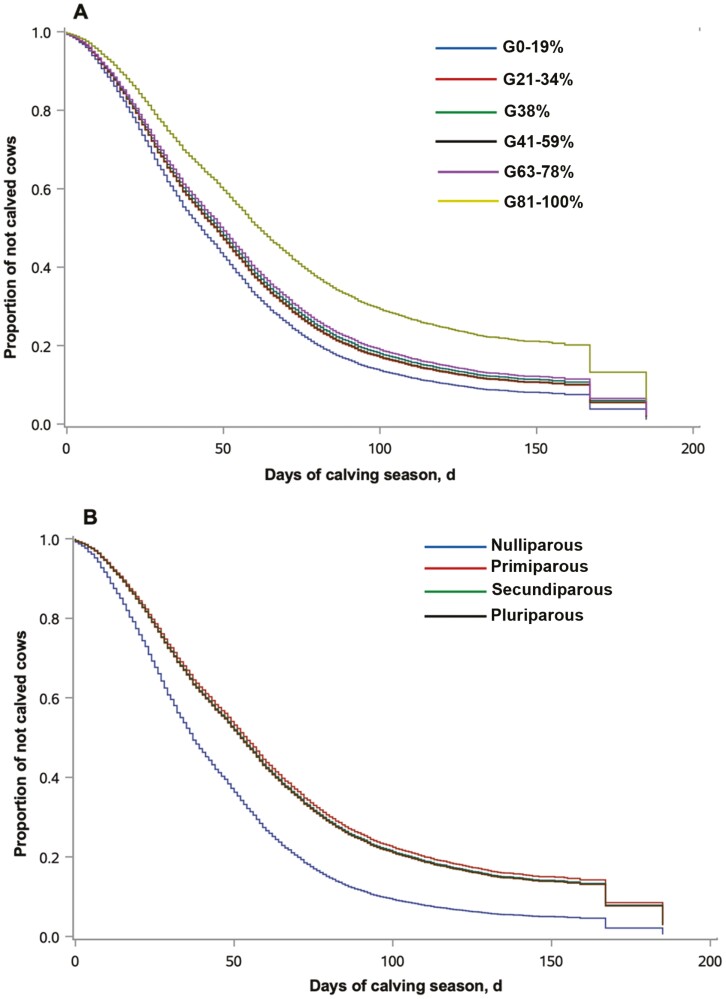
Kaplan–Meier survival curves for calving day up to 185 d of calving seasons according to the (A) proportion of Brahman genetics of Brahman–Angus cows and (B) parity category order of cows. Both, breed group and parity order were associated with rate of calving (*P <* 0.0001). Calving results of 31-yr calving seasons containing the six contemporaneous breed groups were compiled and analyzed (*n* = 6,312). The breed groups within parity orders were exposed to reproduction contemporaneously. Cows in the G81% to 100% took the longest interval to calve. Nulliparous cows took the shortest time to calve because their breeding seasons started 15 to 21 d earlier than the breeding seasons of other parity orders.

The proportion of Brahman genetics and parity order of cows were associated (*P <* 0.0001) with calving distribution. Cows in the G81% to 100% took the longest interval to calve (Fig. 1A and Table 1). According to the AHR, cows in the G0% to 19% had a 69% increase in the rate of calving compared to cows in the G81% to 100%. As such, 50% of cows in the G0% to 19% calved during the first 38 d of the calving seasons, whereas cows in the G81% to 100% only reached this proportion on day 68 of the calving seasons (Table 1). Nulliparous cows took less time to calve during the calving seasons compared with pluriparous cows (Fig. 1B and Table 2), because they entered the breeding season earlier. The rate of calving in the calving seasons was 58 to 62% faster for nulliparous cows than all other three parity groups. Fifty percent of the nulliparous cows calved in the first 34 d of the calving seasons, whereas for the other three parity orders, the median days to calving ranged from 53 to 54 (Table 2).

**Table 1.** Association between the proportion of Brahman genetics of Brahman–Angus cows and rate of calving in calving seasons

Item	Proportion of Brahman genetics						*P* value
	0% to 19%	21% to 34%	38% (Brangus)	41% to 59%	63% to 78%	81% to 100%	
Females, no.	1,180	1,039	876	1,395	848	974	.
Rate of calving^1^							.
AHR (95% CI)^2^	1.69* (1.54 to 1.86)	1.49* (1.35 to 1.64)	1.44* (1.30 to 1.59)	1.48* (1.35 to 1.62)	1.39* (1.26 to 1.54)	1.0 (reference)	<0.0001
Days to calving							
Mean ± SEM	52.3 ± 1.2	59.0 ± 1.4	57.6 ± 1.4	57.3 ± 1.1	60.3 ± 1.4	78.0 ± 1.6	.
Median (95% CI)	38 (36 to 41)	47 (42 to 50)	48 (44 to 51)	47 (45 to 49)	50 (47 to 53)	68 (64 to 71)	.

Within a row, column means with an asterisk differ from the reference group (*P <* 0.001).

^1^A calving season started based on the day the first calf was born in a season (day 0), and the rate of calving was based on the interval between each calving date and day 0.

^2^AHR = Adjusted hazard ratio and confidence interval (CI).

**Table 2.** Association between parity order of cows and rate of calving in calving seasons

Item	Parity order				*P* value
	Nulliparous	Primiparous	Secundiparous	Pluriparous	
Females, no.	1,385	1,422	953	2,552	.
Rate of calving^1^					.
AHR (95% CI)^2^	1.58* (1.47 to 1.70)	0.96 (0.89 to 1.03)	0.99 (0.91 to 1.08)	1.0 (reference)	<0.0001
Days to calving					
Mean ± SEM	49.8 ± 1.3	65.8 ± 1.2	63.8 ± 1.4	66.7 ± 1.2	.
Median (95% CI)	34 (31 to 37)	54 (51 to 57)	53 (49 to 55)	53 (51 to 55)	.

Parity **= **2-yr-old nulliparous, 3-yr-old primiparous, 4-yr-old secundiparous, or pluriparous cows in the previous breeding season.

Within a row, column means with an asterisk differ from the reference group (*P <* 0.001).

^1^A calving season started based on the day the first calf was born in a season (day 0), and the rate of calving was based on the interval between each calving date and day 0.

^2^AHR = Adjusted hazard ratio and confidence interval (CI).

### Association between the proportion of Brahman genetics of Brahman–Angus cows and calf weights

The birth weight of calves was affected (*P *< 0.0001) by breed group, parity order, and calf sex variables. Cows in the G21% to 34%, G38%, and G41% to 59% calved heavier calves than cows in the G0% to 19%, G63% to 78%, and G81% to 100% (Table 3), whereas cows in the G63% to 78% and G81% to 100% calved the lightest calves among the six-breed groups. Nulliparous and primiparous cows calved lighter calves than pluriparous cows, whereas calves from secundiparous cows had birth weights that were intermediate between primiparous and pluriparous cows, but heavier than nulliparous cows (Table 4). At birth, male calves were heavier than female calves (34.2 kg vs. 31.7 kg; SEM: 0.24).

**Table 3.** Association between the proportion of Brahman genetics of Brahman–Angus cows and weight of calves

Item^1^	Proportion of Brahman genetics						SEM	*P* value
	0% to 19%	21% to 34%	38% (Brangus)	41% to 59%	63% to 78%	81% to 100%		
BW, kg	33.1^b^	33.9^a^	33.9^a^	33.4^a,b^	31.8^c^	31.8^c^	0.29	<0.0001
WW, kg	245.2^b^	250.2^a^	247.9^a,b,Y^	252.5^a,X^	245.2^b^	220.6^c^	2.80	<0.0001
205-d WW, kg	215.3^b^	223.6^a^	221.5^a,Y^	225.0^a,X^	222.5^a^	208.9^c^	2.35	<0.0001

Values without a common lowercase (^a,b,c,d^) or uppercase (^X,Y^) superscript in the row differ (*P* < 0.05) or tend to differ (*P* < 0.10), respectively, after applying the Tukey test for multiple comparisons.

^1^BW = birth weight; WW = weaning weight; 205-d WW = weaning weight adjusted to 205-d of age.

**Table 4.** Association between parity order of cows and weight of calves

Item^1^	Parity order				SEM	*P* value
	Nulliparous	Primiparous	Secundiparous	Pluriparous		
BW, kg	31.9^c^	32.8^b^	33.4^a,b^	33.8^a^	0.27	<0.0001
WW, kg	246.1^a,b,X^	239.7^c^	242.2^b,c,Y^	246.5^a^	2.73	<0.0001
205-d WW, kg	209.8^d^	218.4^c^	222.5^b^	227.0^a^	2.29	<0.0001

Parity **= **2-yr-old nulliparous, 3-yr-old primiparous, 4-yr-old secundiparous, or pluriparous cows in the previous breeding season.

^1^BW = birth weight; WW = weaning weight; 205-d WW = weaning weight adjusted to 205-d of age.

**Fig. 2.**
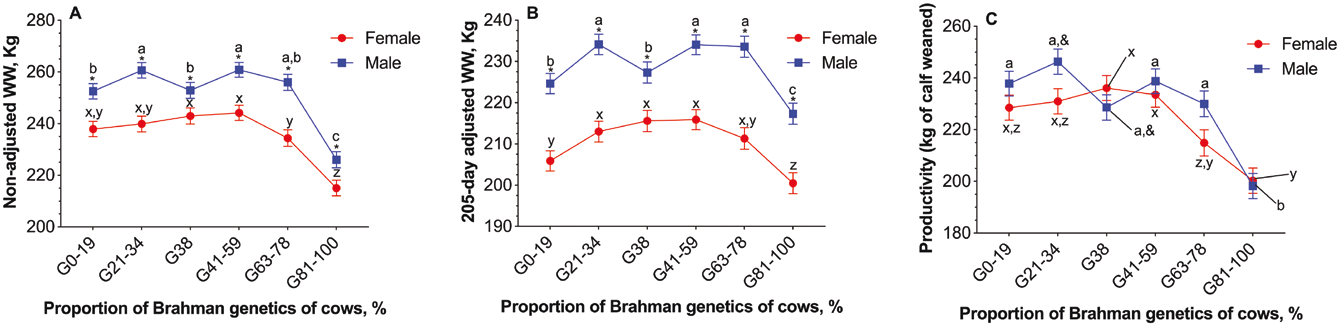
Association between the proportion of Brahman genetics of Brahman–Angus cows (*n* = 5,354) and (A) non-adjusted weaning weight of calves, (B) 205-d adjusted weaning weight of calves, and (C) kilograms of calves weaned per cow exposed to reproduction (productivity), according to calf sex. In all cases, there was an breed group by calf sex interaction effect (*P* < 0.0001) on these variables. The interactions indicated that the association between breed group and weight of calves or productivity varied according to calf sex. Values without a common lowercase within male (^a,b,c^) or female (^x,y,z^) calf categories differ (*P* < 0.05), after applying the Tukey test for multiple comparisons. Values with the common symbol  (^&^) tended to differ (*P* < 0.10). The asterisk indicates significant differences (*P* < 0.05) between male and female calves within each breed group.

**Fig. 3.**
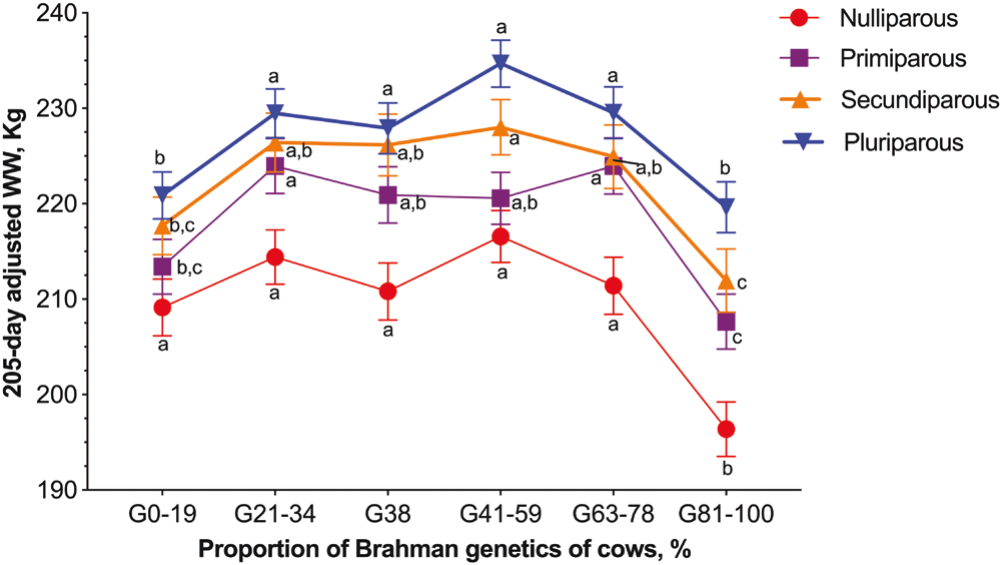
Association between the proportion of Brahman genetics of Brahman–Angus cows (*n* = 5,354) and 205-d adjusted weaning weight of calves according to parity order of cows. There was a breed group by parity order interaction effect (*P* = 0.0618) on 205-d weaning weights. The interaction indicated that the association between breed group and 205-d adjusted weaning weight of calves varied according to parity order. Cows in the G81% to 100% always weaned the lightest calves. Values without a common lowercase (^a,b,c^) within each parity order differ (*P* < 0.05), after applying the Tukey test for multiple comparisons.

The non-adjusted weaning weight of calves (*P* < 0.0001) was affected by the interaction between breed group and calf sex, and by parity order. The two-way interaction revealed that the relationship between breed group of cows and non-adjusted weaning weights varied according to the calf sex (Fig. 2A). Among male calves, cows in the G0% to 19%, G38%, and G81% to 100% weaned the lightest calves. Whereas among female calves, cows in the G63% to 78% and G81% to 100% were the ones weaning the lightest calves. Despite this interaction, male calves were weaned heavier (*P* < 0.0001) than female calves across all breed groups. The non-adjusted weaning weight of calves was 251.5 ± 2.6 kg for males and 235.7 ± 2.6 kg for females. Table 3 depicts the non-adjusted weaning weights of calves. Parity order was associated with weaning weight of calves and pluriparous cows weaned heavier calves than primiparous and secundiparous cows, but not than nulliparous cows (Fig. 3 and Table 4).

The 205-d adjusted weaning weight of calves was affected by the interactions between breed group and calf sex (*P* = 0.0025) and breed group and parity order (*P* = 0.0618). Among male calves, cows in the G0% to 19%, G38%, and G81% to 100% weaned the lightest calves (Fig. 2B). Whereas among female calves, cows in the G0% to 19% and G81% to 100% were the ones weaning the lightest calves. Despite this interaction, male calves were heavier (*P* < 0.0001) than female calves across all breed groups. The 205-d adjusted weaning weight of calves were 228.5 ± 2.2 kg for males and 210.4 ± 2.2 kg for females. Table 3 depicts the 205-d adjusted weaning weight of calves. The interaction between breed group and parity order revealed that among nulliparous cows, the weaning weight of calves from G0% to 19% did not differ from those of cows with 21% to 78% of Brahman genetics. For every other parity order, calves from cows in the G0% to 19% had smaller to intermediate 205-d weaning weight compared with calves from cows with 21% to 78% of Brahman genetics. Even after adjusting the weaning weights to 205-d, pluriparous cows still weaned the heaviest calves (Table 4).

**Fig. 4.**
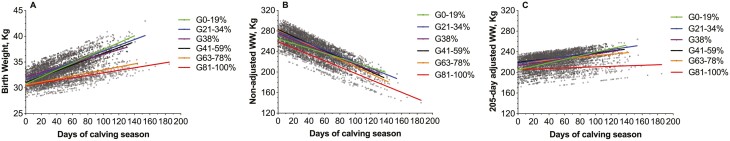
Association between days of calving season and (A) birth weight, (B) non-adjusted weaning weight, and (C) 205-d adjusted weaning weight of calves according to the proportion of Brahman genetics of Brahman–Angus cows. The weight of calves were altered according to the interaction between days of calving season and breed group of cows (*P* < 0.0001). The associations were positive for birth and 205-d adjusted weaning weights, but negative for non-adjusted weaning weight.

When day of the calving season was included in the statistical analyses, then interactions between breed group and day of calving season were detected for birth weight (*P* = 0.0003), non-adjusted weaning weight (*P* < 0.0001), and 205-d adjusted weaning weight (*P* = 0.0003). The regression lines depict positive associations between day of the calving season and birth weight (Fig. 4A) and 205-d adjusted weaning weight (Fig.4C), but a negative association for the non-adjusted weaning weight (Fig. 4B).

### Productivity of Brahman–Angus cows with different proportion of Brahman genetics at weaning

The risk of stillbirth was associated with parity order (*P* = 0.0001) and calf sex (*P* = 0.09). Despite the limited incidence, the risk of stillbirth calves in nulliparous cows was five times greater than any other parity order (Table 6). Also, the risk of stillbirth although rare, it was 1.67 times greater among male calves (0.05%) than female calves (0.03%). The risk of preweaning calf mortality differed (*P *< 0.0001) according to the breed group (Table 5), parity order of cows (Table 6) and calf sex. Cows in the G81% to 100% had twice the preweaning calf mortality than cows with 0 to 59% of Brahman genetics, being intermediate for cows in the G63% to 78%. Preweaning calf mortality among nulliparous cows was twice that of cows from other parity orders. A larger proportion of male calves (7.6%) died preweaning compared with female calves (4.5%).

**Table 5.** Association between the proportion of Brahman genetics and productive traits

Item	Proportion of Brahman genetics						*P* value
	0% to 19%	21% to 34%	38% (Brangus)	41% to 59%	63% to 78%	81% to 100%	
Preg. loss^1^, %	0.6	0.3	1.3	0.7	0.6	1.2	.
(no./no.)	(6/1,020)	(3/889)	(10/749)	(8/1,189)	(4/729)	(10/829)	
Calving^2^, %	85.9	85.3	84.4	84.7	85.5	84.1	.
(no./no.)	(1,014/1,180)	(886/1,039)	(739/876)	(1,181/1,395)	(725/848)	(819/974)	
Stillbirth^3^, %	0.04	0.03	0.06	0.04	0.04	0.05	0.57
(no./no.)	(19/1,014)	(9/886)	(17/739)	(18/1,181)	(13/725)	(17/819)	
Calf mortality^4^,%	4.3^b^	4.3^b^	5.7^a,b,Y^	5.8^b^	7.0^a,b^	9.2^a,X^	<0.0001
(no./no.)	(54/1,014)	(44/886)	(50/739)	(85/1,181)	(59/725)	(90/819)	
Weaning^5^, %	95.7^a^	95.7^a^	94.3^a,b,X^	94.2^a^	93.0^a,b^	90.8^b,Y^	<0.0001
(no./no.)	(960/1,180)	(842/1,039)	(689/876)	(1096/1,395)	(666/848)	(729/974)	
Non-adjusted productivity^6^, kg/cow	233.2^a,b,X^ ± 3.9	238.6^a^ ± 4.0	232.4^a,b^ ± 4.0	236.1^a^ ± 3.9	222.4^b,Y^ ± 4.0	199.3^c^ ± 3.9	<0.0001
Adjusted productivity^7^, kg/cow	191.1^b,Y^ ± 2.3	197.0^a^ ± 2.3	195.9^a,b,X^ ± 2.3	199.7^a^ ± 2.3	196.2^a,b,X^ ± 2.3	181.8^c^ ± 2.3	<0.0001

^1^Pregnancy loss: proportion of cows that never calved in a given calving season out of the pregnant cows expected to calve.

^2^Calving: proportion of cows exposed to reproduction that calved during the calving season.

^3^Stillbirth: LSMEANS of proportion of stillbirth calves.

^4^Calf mortality: LSMEANS of proportion of calves that died between birth and weaning.

^5^Weaning rate: LSMEANS of proportion of cows weaning a calf.

^6^Non-adjusted productivity: LSMEANS of kilograms of calf weaned per cow exposed to reproduction non-adjusted for weaning rate nor age of calf at weaning.

^7^Adjusted productivity: LSMEANS of kilograms of calf weaned per cow exposed to reproduction adjusted for weaning rate and age of calf at weaning.

Values without a common lowercase (^a,b,c^) or uppercase (^X,Y,Z^) superscript in the row differ (*P <* 0.01) or tend to differ (*P <* 0.10), respectively, after applying the Tukey test for multiple comparisons.

**Table 6.** Association between parity order of cows and productive traits

Item	Parity order				*P* value
	Nulliparous	Primiparous	Secundiparous	Pluriparous	
Preg. loss^1^, %	0.6	1.5	0.1	0.7	.
(no./no.)	(7/1,222)	(17/1,173)	(1/811)	(16/2,199)	
Calving^2^, %	87.7	81.3	85.0	85.5	.
(no./no.)	(1,215/1,385)	(1,156/1,422)	(810/953)	(2,183/2,552)	
Stillbirth^3^, %	0.1^a^	0.02^b^	0.02^b^	0.04^b^	<0.0001
(no./no.)	(39/1,215)	(11/1,156)	(7/810)	(36/2,183)	
Calf mortality^4^, %	8.5^a^	4.8^b^	4.7^b^	6.1^b^	0.0009
(no./no.)	(121/1,215)	(67/1,156)	(42/810)	(152/2,183)	
Weaning^5^, %	91.5^b,Y^	95.2^a^	95.4^a^	93.9^a,b,X^	0.0009
(no./no.)	(1,094/1,385)	(1,089/1,422)	(768/953)	(2,031/2,552)	
Non-adjusted productivity^6^, kg/cow	224.4 ± 3.9	226.4 ± 3.6	229.0 ± 3.6	228.3 ± 3.5	0.58
Adjusted productivity^7^, kg/cow	188.3^c^ ± 2.4	192.1^b,c^ ± 2.1	195.2^b^ ± 2.1	198.9^a^ ± 2.1	<0.0001

Parity **= **2-yr-old nulliparous, 3-yr-old primiparous, 4-yr-old secundiparous, or pluriparous cows in the previous breeding season.

^1^Pregnancy loss: proportion of cows that never calved in a given calving season out of the pregnant cows expected to calve.

^2^Calving: Proportion of cows exposed to reproduction that calved during the calving season.

^3^Stillbirth: LSMEANS of proportion of stillbirth calves.

^4^Calf mortality: LSMEANS of proportion of calves that died between birth and weaning.

^5^Weaning rate: LSMEANS of proportion of cows weaning a calf.

^6^Non-adjusted productivity: LSMEANS of kilograms of calf weaned per cow exposed to reproduction non-adjusted for weaning rate nor age of calf at weaning.

^7^Adjusted productivity: LSMEANS of kilograms of calf weaned per cow exposed to reproduction adjusted for weaning rate and age of calf at weaning.

Values without a common lowercase (^a,b,c^) or uppercase (^X,Y^) superscript in the row differ (*P <* 0.05) or tend to differ (*P* < 0.10), respectively, after applying the Tukey test for multiple comparisons.

The weaning rate differed (*P* < 0.0001) according to the breed group, parity order and calf sex, independently. Cows in the G81% to 100% weaned fewer calves than cows with 0% to 59% of Brahman genetics (Table 5). Cows in the G63% to 78% had intermediate weaning rates. Nulliparous cows weaned the fewest number of calves (Table 6), and fewer bull calves (92.4%) were weaned when compared to female calves (95.5%).

The non-adjusted productivity of cows was altered (*P* = 0.02) by the interaction between breed group and calf sex (Fig. 2C). Cows in the G81% to 100% were the least productive cows whether they calved a male or female calf. For the other breed groups, nuances existed in the productivity according to calf sex. Among male calves, cows with 0% to 78% of Brahman genetics had comparable productivity. Whereas among female calves, cows in the G38% and G41% to 59% were the most productive. In general, cows weaning male calves were more productivity than those weaning female calves (230.0 vs. 224.1; SEM: 3.19) and cows in the G21% to 34% and G41% to 59% were the most productive cows (Table 5).

After adjusting productivity for age of calves at weaning and weaning rate, the adjusted productivity was altered (*P* < 0.0001) according to breed group (Table 5) and parity order (Table 6) of cows. Cows in the G81% to 100% continued to be the least productive. Cows in the G0% to 19% were more productive than cows in G81% to 100%, but less productive than cows with 21% to 78% of Brahman genetics. Nulliparous cows were the least productive of the parity groups.

## Discussion

In this study, the productivity of Brahman–Angus cows was evaluated according to the proportion (i.e., 0% to 100%) of the cows’ Brahman genetics. Cows with 81% to 100% of Brahman genetics calved at slower rate than cows with 0% to 78% of Brahman genetics, weaned the lightest calves, and had the greatest preweaning calf mortality. Cows with 81% to 100% of Brahman genetics were the least productive cows even after adjusting for age of calves at weaning and weaning rates. Despite the fact that cows with 0% to 19% of Brahman genetics calved at faster rate and had smaller preweaning calf mortality than cows with 81% to 100% of Brahman genetics, they weaned lighter calves than cows with 21% to 78% of Brahman genetics. Even after adjusting for the age of calves at weaning and weaning rates, cows with 0% to 19% of Brahman genetics remained less productive. Thus, a proportion of Brahman genetics between 21% and 78% ensured superior productivity of Brahman–Angus cows subjected to subtropical conditions.

Number of kilograms of calves produced per cow submitted to reproduction is a product between the number and weight of calves weaned. Number of calves produced was similar across different parity orders and breed groups, but cows in the G81% to 100% weaned fewer calves than any other breed group due to the high incidence of preweaning calf mortality. As cows calved during the winter, calves with greater proportion of Brahman genetics may have suffered with cold stress, predisposing cows in the G81% to 100% to lose more calves. In the North-Central Florida, despite winter being mild, freezing temperatures are commonly observed. Cold-stressed neonatal Brahman calves could not regulate their body temperature as well as ½ Simmental × ¼ Brahman × ¼ Hereford calves (Godfrey et al., 1991). The authors also verified that the peripheral concentration of blood energy-containing constituents, such as glucose, fatty acids, and triglycerides, were greater in cold-stressed Brahman calves. This suggested that cold-stressed Brahman calves could not effectively mobilize the blood energy constituents to regulate their body temperature. Brahman calves are also susceptible to weak calf syndrome (Roberson et al., 1986; Carstens, 1994). Newborn calves with this syndrome display muscular weakness, take longer to stand for the first time, and lack a strong suckle reflex. The causes of weak calf syndrome still unclear, but the greater frequency in Brahman cattle suggests a genetic component. Thus, the productivity of cows in the G81% to 100% was compromised by the high incidence of preweaning calf mortality when these cows were calving during subtropical winter months, when freezing temperatures were observed. Even after adjusting for weaning rates, cows in the G81% to 100% remained among the least productive, indicating that genetics itself also undermined the production of these cows.

Within parity order, nulliparous cow had the greatest proportion of preweaning calf mortality. Nulliparous cows calving as 2- and 3-yr-old accounted for 41% of the overall 6.7% calf death in an observational study with 13,296 calving records (Patterson et al., 1987). Abortion, stillbirth, calf abnormalities, and abandonment were within the most common causes of calf death in nulliparous cows (Bellows et al., 1987; Patterson et al., 1987). Similarly, herein, nulliparous cows had up to five times greater proportion of stillbirth than older cows, negatively impacting the weaning rates.

Weaning weight is an important component affecting productivity of cows. The association between proportion of Brahman genetics of cows and weaning weight of calves resembled a quadratic relationship. In general, cows with somewhat moderate to slightly high proportion of Brahman genetics (21% to 78%) weaned heavier calves than cows with very low (0% to 19%) or high (81% to 100%) proportion of Brahman genetics. This association was even more evident after adjusting the weaning weight of calves to 205-d. The lack of hybrid vigor among a few calves from the cows in the G0% to 19% and G81% to 100% likely contributed to these results, as heterosis favors the productivity of *B. indicus* and *B. taurus* crossbred cows over *B. indicus* purebred cows in the tropics (Trail et al., 1985; Dominguez-Castaño et al., 2021). It was also possible that the nurturing ability of cows varied according to the proportion of Brahman genetics. For example, *B. indicus* purebred cows produced less milk than *B. taurus* purebred cows (Syrstad, 1985; Galukande et al., 2013). Thus, cows in the G81% to 100% could have produced less milk, limiting the daily body weight gain of calves. Cows in the G81% to 100% also calved at slower rate as a reflex of prolonged time to conceive during the breeding season and longer gestation length (Martins et al., 2022). It is well known that cows conceiving earlier wean older and heavier calves (Rodgers et al., 2012; Cushman et al., 2013). In this sense, part of the inefficiencies of cows in the G0% to 19% was minimized because they calved at a faster rate. The 205-d adjusted weaning weight, nevertheless, evidenced the differences on weaning weights of calves from this group of cows. Cows in the G0% to 19% as well as their calves may have suffered to cope with heat-stress, compromising the performance of cow–calf pairs. An earlier study demonstrated that the 2-yr-old Brahman–Angus heifers with 0% proportion of Brahman genetics had the lowest heat-stress resilience during summer days while on pasture in Florida (Mateescu et al., 2020). Heifers with 75% to 100% and 25% to 50% of Brahman genetics had superior to intermediate heat-stress resilience, respectively. The productivity analyses followed the same trend as the weaning weight analyses, even after adjusting for weaning rates and age of calves at weaning. Thus, indicating that the genotype of the cows itself accounted for the differences in calves’ weaning weight and productivity of cows.

The associations between breed group of cows and weaning weight of calves and between breed group and productivity depended on calf sex and parity order of cows. Regarding to the calf sex, male calves were always heavier than female calves as verified by other authors studying population of crossbred cattle (Casas et al., 2011). The breed group by calf sex interaction effects indicated that among female calves, the influence of breed group on calf weaning weight or productivity was not as evident as among male calves. This two-way interaction effect suggested that the female calves were less susceptible to the variation of proportion of Brahman genetics of cows, probably due to the genetic differences between male and female calves. The breed group by parity order interaction effect indicated that among nulliparous cows, the influence of breed group on 205-d adjusted weaning weight was not as evident as among the other parity orders. Additionally, among pluriparous cows, cows in the G81% to 100% did not wean the lightest calves; instead, the 205-d adjusted weaning weight was comparable to the cows in the G0% to 19%. Thus, it seems that maturity of cows attenuated or compensated for some of the inefficiencies related to the proportion of Brahman genetics of cows. In general, pluriparous cows weaned heavier calves and were more productive than nulliparous, primiparous, and secundiparous cows. Nulliparous cows weaned the lightest calves after adjusting the weaning weight of calves to 205-d. Besides lactation and gestation, younger cows have extra nutritional requirements for growing (Short et al., 1990; Ferreira et al., 2021). In this present study, it is possible that the nutritional management of nulliparous, primiparous, and secundiparous cows were sub-optimal, compromising their performance as dams. Thus, in general, cows with 0% to 19% and 81% to 100% of Brahman genetics were less productive than cows with 21% to 78% of Brahman genetics. However, the extent of difference on productivity among these group of cows varied according to calf sex and parity order of cows.

The later the cows calved, the greater was the birth weight and the 205-d adjusted weaning weight of calves. Conversely, the non-adjusted weaning weights were associated negatively with calving dates. These contradictory results can be related to the changes in nutritional management of the herd during the winter, when cows received some supplementation and grazed good quality cool-season grasses. The early calving cows may have experienced some level of nutritional restriction during the last trimester of pregnancy before the implementation of the winter nutritional regimen. The fetus undergoes rapid growth during mid-to-late gestation which increases the nutritional requirements for pregnancy (Ferrell et al., 1976; Hoffman et al., 2017). Cows undergoing restriction at this phase calved lighter calves (Larson et al., 2009; Bohnert et al., 2013; Funston and Summers, 2013). The nutritional restriction at the last trimester of pregnancy also impacted negatively the performance of steers and heifers postnatally (Larson et al., 2009; Funston et al., 2010). Thus, the non-supplemented and early calving cows probably experienced greater energy deficits than the winter-supplemented and later calving cows, explaining the birth weight and 205-d adjusted weaning weight results.

Collectively, subtropical Brahman–Angus cows with 21% to 78% of Brahman genetics were more productive. On the one hand, the cows with 81% to 100% of Brahman genetics were the least productive because of more calves died before weaning and they weaned lighter calves. The susceptibility of Brahman calves to cold stress is one explanatory factor for the elevated incidence of preweaning calf mortality, as cows calved during winter months amidst freezing temperatures. On the other hand, cows with 0% to 19% of Brahman genetics were under productive because they weaned lighter calves. This group of cows may have been less able to cope with the harsh subtropical environmental conditions, such as heat-stress, compromising their performance as dams. Younger cows weaned lighter calves than old and mature pluriparous cows. Male calves were also heavier at weaning than female calves. These two secondary factors modulated the extent of influence of proportion of Brahman genetics of cows on calf weaning weight. Overall, moderate to slightly high *B. indicus* genetics (21% to 78%) ensured greater productivity of subtropical Brahman–Angus cows.
